# Using machine learning to predict gamma passing rate in volumetric‐modulated arc therapy treatment plans

**DOI:** 10.1002/acm2.13824

**Published:** 2022-12-09

**Authors:** Elahheh Salari, Kevin Shuai Xu, Nicholas Niven Sperling, E. Ishmael Parsai

**Affiliations:** ^1^ Department of Radiation Oncology University of Toledo Medical Center Toledo Ohio USA; ^2^ Department of Computer and Data Sciences Case Western Reserve University Cleveland Ohio USA

**Keywords:** gamma passing rate, machine learning, pretreatment verification, VMAT

## Abstract

**Purpose:**

This study aims to develop an algorithm to predict gamma passing rate (GPR) in the volumetric‐modulated arc therapy (VMAT) technique.

**Materials and methods:**

A total of 118 clinical VMAT plans, including 28 mediastina, 25 head and neck, 40 brains intensity‐modulated radiosurgery, and 25 prostate cases, were created in RayStation treatment planning system for Edge and TrueBeam linacs. In‐house scripts were developed to compute Modulation indices such as plan‐averaged beam area (PA), plan‐averaged beam irregularity (PI), total monitor unit (MU), leaf travel/arc length, mean dose rate variation, and mean gantry speed variation. Pretreatment verifications were performed on ArcCHECK phantom with SNC software. GPR was calculated with 3%/2 mm and 10% threshold. The dataset was randomly split into a training (70%) and a test (30%) dataset. A random forest regression (RFR) model and support vector regression (SVR) with linear kernel were trained to predict GPR using the complexity metrics as input. The prediction performance was evaluated by calculating the mean absolute error (MAE), *R* ^2^, and root mean square error (RMSE).

**Results:**

RMSEs at *γ* 3%/2 mm for RFR and SVR were 1.407 ± 0.103 and 1.447 ± 0.121, respectively. MAE was 1.14 ± 0.084 for RFR and 1.101 ± 0.09 for SVR. *R* ^2^ was equal to 0.703 ± 0.027 and 0.689 ± 0.053 for RFR and SVR, respectively. GPR of 3%/2 mm with a 10% threshold can be predicted with an error smaller than 3% for 94% of plans using RFR and SVR models. The most important metrics that had the greatest impact on how accurately GPR can be predicted were determined to be the PA, PI, and total MU.

**Conclusion:**

In terms of its prediction values and errors, SVR (linear) appeared to be comparable with RFR for this dataset. Based on our results, the PA, PI, and total MU calculations may be useful in guiding VMAT plan evaluation and ultimately reducing uncertainties in planning and radiation delivery.

## 1 INTRODUCTION

Over the last two decades, the complexity of external beam radiation therapy has increased significantly. Volumetric‐modulated arc therapy (VMAT) often requires more beam intensity modulation, which typically results in a higher degree of complexity and larger dosimetric uncertainty. Consequently, pretreatment verification before treatments is highly recommended to ensure safe delivery.

For comparison between the calculated and measured dose distributions, the gamma index is commonly used, which combines both percentage dose difference and distance‐to‐agreement. According to the American Association of Physicists in Medicine (AAPM) Task Group 218 (TG‐218), 3%/2 mm with a 95% passing rate is recommended.^1^ However, the VMAT planning and delivery are subject to many sources of error and uncertainty which may reduce the gamma passing rate (GPR) below 95%.

Many studies using different treatment planning systems (TPS) and linacs have shown that the degree of plan complexity may affect the accuracy of dose calculations and treatment delivery,^2^, ^3^, ^4^, ^5^, ^6^ which is crucial in dosimetry audits and clinical trials. Hence, different aspects of treatment such as the quality and complexity of treatment plans have to be carefully evaluated.

Recently, machine‐learning (ML) and deep learning methods have been widely used to predict patient‐specific QA results. Granville et al. trained a support vector classifier with a linear kernel to predict VMAT patient QA results.^7^ They performed all patient‐specific QAs on a diode‐array detector and used both treatment plan complexity and linac performance metrics to train their model.^7^ In 2018, Nyflot et al.^8^ used deep learning with convolutional neural networks that include radiomics to predict and find underlying errors related to patient‐specific QA using an electronic portal imaging device. In this study, no complexity metrics were included. Tomori et al.^9^ conducted a study on 60 prostate cases with a convolutional neural network to predict GPR using Gafchromic EBT3 films. Sagittal plane dose distribution, target and organ‐at‐risk volumes, and monitor unit (MU) were considered independent variables to build their model. In 2019, Ono et al.^10^ used complexity metrics such as PA, PI, and modulation complexity score (MCS), to train three ML algorithms (regression tree analysis, multiple regression analysis, and neural network) using ArcCHECK phantom for QAs to predict passing rate.

According to previous studies, ML has been shown to be useful for predicting passing rates. Based on our literature review, support vector regression (SVR) with linear kernel was not used before for predicting GPR values. Consequently, in this study, we used SVR with the linear kernel to predict the values of GPR and compare it with random forest regression (RFR), whereby its accuracy for GPR prediction has been shown in previous literature.^11^, ^12^ Different modulation indices (MI), which have been previously developed, were utilized to build both models.

## 2 MATERIALS AND METHODS

### 2.1 Treatment planning

A mix of VMAT treatment sites consisting of a total of 118 clinical plans were randomly selected for this study. All treatment plans were created in RayStation version 10.A (RaySearch Medical Laboratories AB, Stockholm, Sweden) TPS with a grid size of 1 mm for intensity‐modulated radiosurgery (IMRS) cases and 2 mm for other treatment sites. The dose‐calculation algorithm was collapsed‐cone convolution version 5.3, and gantry angle sampling of 2° between the control points was used for all the optimized plans. Two Varian linacs (Edge and TrueBeam‐Varian Medical Systems, Palo Alto, CA) with 6 MV‐FFF, 6 MV, and 10 MV beams were utilized. Edge linac is equipped with high definition multileaf collimator (HD‐120 MLC), and TrueBeam has standard Millennium 120 MLC. A summary of the treatment plans showing beam energy, the number of beams, and the linac used for treatment are shown in Table 1. Depending on the geometry and the location of the targets, the number of arcs or partial arcs varied per plan.

**TABLE 1:** The number of plans for each machine and energy

	IMRS	H&N	Med/lung	Prostate	Total
TrueBeam	–	25	16	25	66
Edge	40	–	12	–	52
6 MV‐FFF	40	21	24	–	85
6 MV	–	4	4	–	8
10 MV	–	–	–	25	25
No. beam	236	92	72	76	476
Technique	SIMT	VMAT	VMAT/SBRT	VMAT	

Abbreviations: FFF, flattening filter free; H&N, head and neck; IMRS, intensity‐modulated radiosurgery; Med, mediastinum; SBRT, stereotactic body radiation therapy; SIMT, single isocenter multiple target; VMAT, volumetric‐modulated arc therapy.

### 2.2 Second MU check and pretreatment verification

VMAT technique requires more beam intensity modulation, which usually results in a larger number of MUs. Moreover, uncertainties in using CT/MR/PET simulation, inhomogeneity corrections, and complex calculation algorithms such as convolution/superposition and Monte Carlo warrant the need of doing a second MU check to assure the accuracy of dose calculations.^13^ Hence, each plan received an independent MU check by using RadCalc (Life‐Line Software, Inc., LAP Group, Ver6.4).

Next, plan delivery accuracy was measured using an ArcCHECK (Sun Nuclear Corporation, Melbourne, FL) phantom and the SNC‐Patient software (Ver 8.2) following a modified true composite setup as presented in the TG‐218 report of AAPM^1^ using a field‐by‐field in treatment position instead of composite evaluation only. The gamma index was calculated using 3%/2 mm and a 10% threshold as per recommendations in TG‐218.^1^ GPR was calculated using a global normalization for the absolute dose, resulting in higher GPR due to reducing the percentage contribution of number of failing points.^14^

### 2.3 Modulation indices

We developed several Python scripts to compute complexity metrics consisting of the following components for each segment: The aperture area was calculated as the total area of all MLC openings; the aperture perimeter (AP) is the length of the boundary of the total area of all MLC openings; and the aperture irregularity (AI) represents the deviation of aperture shape from the circle, and it equals one if the aperture is a perfect circle. For each beam, the beam area (BA) is the total area of all MLC openings of the beam, and the beam irregularity (BI) is computed using AI. For each plan, the plan‐averaged beam area (PA) and the plan‐averaged beam irregularity (PI) are calculated by averaging the BA and BI values using the beam MUs as weighting factors.^15^ leaf travel (LT)^16^ was also calculated. As was demonstrated by other studies,^2^, ^17^ PI and modulated complexity score (MCS), which is represented by McNiven et al.,^18^ is a complex algorithm to predict the deliverability and the accuracy of IMRT plans by using positions of leaf, degree of irregularity in field shape, the weight of the segment, and area. And also edge metric described by Younge et al.^19^ quantifies the complexity of MLC aperture based on the ratio between the MLC AP and area. All provide similar information; therefore, only PI was calculated in this study.

Moreover, LT/arc length (AL) was calculated due to LT being originally formulated for the VMAT plan with one single arc.^16^ As a result of a comparison between plans with a different number of arcs or partial arcs, the average distance traveled by MLC inside the fields over the VMAT arc was divided by AL.^2^ Mean dose rate (DR) variation: the sum of DR variations overall control points divided by AL ^17^; mean gantry speed (GS) variation: sum of the GS variations overall control points divided by the AL.^17^ Table 2 shows complexity metrics extracted from TPS directly to build the model.

**TABLE 2:** The extracted features used in the random forest regression model

AA	The aperture area
AP	The aperture perimeter
AI	The aperture irregularity
BI	Beam irregularity
BA	Beam aperture area weighted by MU
PI	The plan‐averaged beam irregularity
PA	The plan‐averaged beam area
Leaf travel	The average distance traveled by MLC
Gantry speed	Mean gantry speed variation
Dose rate	Mean dose rate variation
Total MU	Total MU per plan

Abbreviations: MLC, multileaf collimator; MU, monitor unit.

### 2.4 Statistical analysis

There may be complex and unknown relationships between the dependent and independent variables in the dataset. Discovering and quantifying the degree to which variables in the dataset are dependent upon each other is an important step in machine learning because this information can help us better prepare our data to meet the requirements of ML algorithms. The Pearson correlation coefficient (CC) was calculated between MIs and GPR. The CC ranges were defined to be 0.0 < CC < 0.2 for no correlation, 0.21 < CC < 0.4 for weak correlation, 0.41 < CC < 0.6 for moderate correlation, 0.61 < CC < 0.8 for strong correlation, and CC greater or equal to 0.8 for very strong correlation.^20^

Furthermore, one‐way ANOVA was performed to determine whether there are any statistically significant differences among all treatment sites regarding MIs. All statistical analysis was performed using SPSS (Ver.27).

### 2.5 Trajectory log files analysis

It has been indicated that machine log file analysis is a robust and efficient way to detect errors originating from human mistakes, flawed planning, and data transfer problems.^21^, ^22^, ^23^, ^24^ The chance of finding these errors is low using a diode‐array detector (e.g., ArcCHECK phantom) due to low detector density.^21^, ^25^, ^26^

Therefore, a total of 262 and 214 trajectory log files for Edge and TrueBeam, respectively, were analyzed using Pylinac (Ver 3.0) to find any potential errors and ensure the accuracy of plan deliverability. The percentage error was calculated by using the following equation:

(1) $$\%\mathrm{Error}\;=\;\frac{\left|\mathrm{actual}-\mathrm{expected}\right|}{\mathrm{expected}}\times 100$$

where actual is the delivered plan parameters (MU, gantry angle, collimator angle, jaw positions, and MLC positions), and expected is all these parameters calculated by the TPS.

### 2.6 Machine‐learning models

The calculated features were shuffled and then randomly split into 70% (82 treatment plans) being fed into the training dataset and 30% (36 treatment plans) into the test dataset. In this study, the Scikit‐learn implementation of RFR and SVR with linear kernel was used, which gives the user an option for tuning the hyperparameters for both models. To tune hyperparameters and avoid overfitting, a 10‐fold cross‐validation was conducted. Moreover, the permutation‐importance of the Scikit‐learn was used to demonstrate what variable has the most impact on GPR.^27^ The regression models contain six complexity metrics as independent variables, and the dependent variable is the GPR that has to be predicted. The prediction performance was evaluated by calculating the mean absolute error (MAE) and root mean square error (RMSE). MAE and RMSE were computed 30 times for different train and test sets for each model.

#### 2.6.1 Random forest regression

In recent years, one of the ML models that has been used to predict GPR is RFR.^11^, ^12^ RF is a tree‐based model; therefore, it constructs several decision trees (DTs) during training time, and the final prediction is the average of the outputs of the DTs. Moreover, RF is a bagging technique; therefore, all computations are run in parallel, and there is no interaction between DTs. Each of the DT builds a scheme of bagging where a subset of features is randomly selected, and a subset of sample instances is randomly chosen to build the tree,^11^, ^12^, ^28^ which means that no single tree can see all the data. This helps to focus on the general patterns within the training data and reduces sensitivity to noise. The GridSearchCV was executed to tune hyperparameters, including the maximum number of features to be used in a DT, the number of DT, the maximum depth of trees, the minimum sample of splits, and a minimum sample of leaves for RFR (Table 3).

**TABLE 3:** Hyperparameters used to train the random forest regression (RFR) model

*n*‐Estimators	Min‐samples‐leaf	Min‐samples‐split	Max‐features	Max‐depth
150	6	3	Sqrt	80

#### 2.6.2 Support vector regression

SVR is a powerful algorithm that has been used for radiotherapy outcome predictions such as the prediction of respiratory tumor motion,^29^, ^30^ and predicting clinical outcomes after radiotherapy.^31^, ^32^ This model is flexible to define how much error is acceptable and find an appropriate line or hyperplane, potentially in higher dimensions, to fit the data. SVR is a kernel‐based model, which includes different kernels for nonlinear regression. The kernel function generally transforms the training set of data to a higher dimension and then fits the data in the higher dimensional space. Common kernels that have been used widely are the linear kernel, Gaussian kernel radial basis function (RBF), and polynomial (poly) kernel.

In this study, GridSearchCV from the Scikit‐learn package was used to tune hyperparameters, including kernels (linear, RBF, poly), epsilon, C, and gamma. In the case of this dataset, the best parameters were determined to be SVR with linear kernel, *C* = 1, and epsilon = 0.5. In 2006, Crone et al.^33^ conducted a study regarding the impact of preprocessing in time series predictions using SVR with different kernels and neural networks. The study indicated their result can easily be outperformed by using SVR with linear kernel; additionally, its accuracy is comparable with feedforward neural network across all artificial time series patterns.^33^ Moreover, to the best of our knowledge, no one used this algorithm for the prediction of GPR values before. As a result, SVR with a linear kernel was implemented in our study.

## 3 RESULTS

### 3.1 Second MU check and pretreatment verification

All computed dose differences between the RadCalc and TPS were less than 3% for all treatment sites.

**FIGURE 1:**
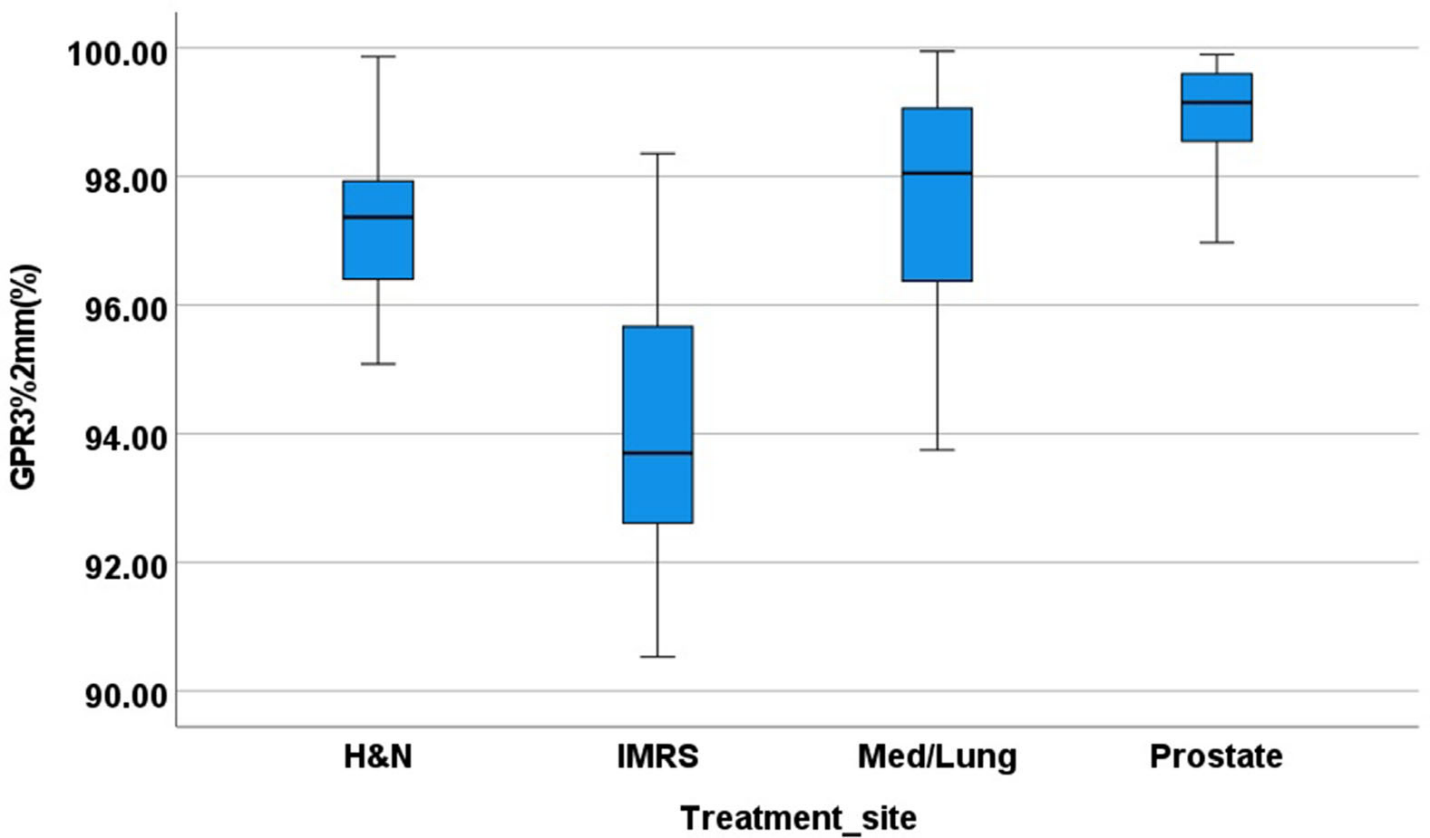
Boxplots for gamma passing rate (GPR) (3%/2 mm) based on treatment site. Intensity‐modulated radiosurgery (IMRS) has the lowest GPR, whereas the prostate has the highest GPR among all treatment sites. This chart shows the distribution of data into quartiles (“minimum,” first quartile (Q1), median (the line within the box), third quartile (Q3), and “maximum”). Med refers to mediastinum.

Figure 1 shows the data related to GPR for each treatment site. As it was shown in Figure 1, IMRS has the lowest GPR among all groups, whereas the prostate has the highest passing rate.

### 3.2 Modulation indices

**FIGURE 2:**
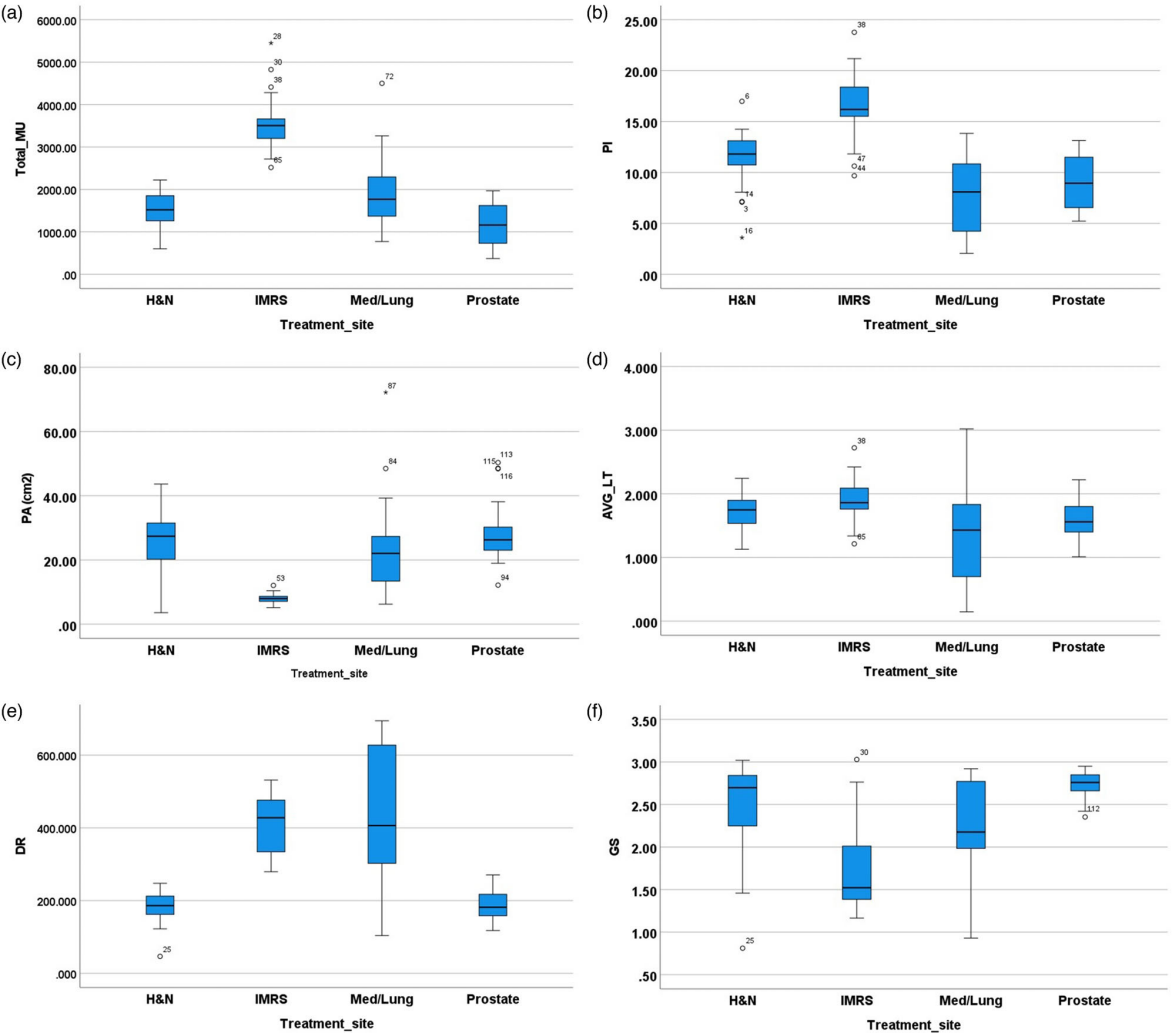
Boxplots of (a) total_MU, (b) plan‐averaged beam irregularity (PI), (c) plan‐averaged beam area (PA), (d) leaf travel per arc length (LT/AL), (e) mean dose rate (DR) variation, and (f) mean gantry speed (GS) variation for head and neck, intensity‐modulated radiosurgery (IMRS), mediastinum/lung, and prostate. This chart shows the distribution of data into quartiles (“minimum,” first quartile [Q1], median [the line within the box], third quartile [Q3], and “maximum”). Dots outside the box are outliers, and Med refers to mediastinum.

Figure 2 shows the beam complexity results obtained from the head and neck, IMRS, mediastinum/lung, and prostate VMAT plans. The data suggested that the complexity metrics were not uniform across different treatment sites, and as shown in Figure 2, IMRS plans have the highest PI values and the lowest PA. The varied complexities are probably due to the special target geometry in each treatment site and the position of normal structure around the target(s). Statistically significant differences in MIs among all treatment sites are shown in Table 4.

**TABLE 4:** Shows the results of one‐way ANOVA among all treatment sites

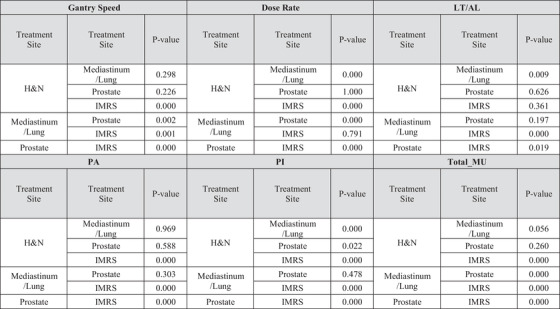

Abbreviations: AL, arc length; H&N, head and neck; IMRS, intensity‐modulated radiosurgery; LT, leaf travel; MU, monitor unit.

### 3.3 Statistical analysis

In careful analysis of computed data, the following results were observed: a strong correlation among GPR and PA, PI, total MU, and GS (0.723, −0.672, −0.66, and 0.624, respectively, with *p* < 0.001), negative moderate correlation between GPR and DR (−0.497) (*p* < 0.001), and negative weak correlation between GPR and LT/AL (−0.382) (*p* < 0.05). Table 4 shows the statistically significant differences among different MIs for all treatment sites.

### 3.4 Trajectory log files analysis

Table 4, 5 demonstrates the maximum percentage error between treatment plans and deliveries for MLC positions, jaws positions, MU, gantry, and collimator angles. As displayed in Table 5, the maximum percentage errors using Equation (1) are less than 0.1% for all treatment sites.

**TABLE 5:** Maximum percentage difference between plan and delivery for each treatment site

	IMRS	H&N	Med/lung	Prostate
MLC position (%)	9.00E − 03	1.05E − 02	1.10E − 02	9.10E − 03
MU (%)	6.60E − 02	6.31E − 02	6.70E − 02	1.80E − 02
Gantry (%)	8.70E − 02	1.75E − 01	5.70E − 02	5.30E − 02
Collimator (%)	3.00E − 05	1.02E − 04	4.00E − 04	2.00E − 04
*X* jaw (%)	7.01E − 04	2.00E − 04	4.00E − 04	8.00E − 04
*Y* jaw (%)	7.00E − 05	7.70E − 04	1.03E − 05	3.70E − 04

Abbreviations: H&N, head and neck; IMRS, intensity‐modulated radiosurgery; MLC, multileaf collimator; MU, monitor unit.

### 3.5 Machine‐learning models

Applying SVR (linear) directly to GPR did not result in good prediction values as some of the prediction values were greater than 100%, which is not acceptable for GPR.

To avoid this issue, a logit transform was applied to GPRs. First, GPRs were divided by 100 to have data in the range between 0 and 1. However, as it is shown in Figure 1, GPRs are concentrated in the range (0.9,1). This is right in most nonlinear portions of the logit function and can result in worse prediction values. Hence, after dividing by 100, data were subtracted by 0.9, and if the results of subtraction were negative, those values were set to zero. After this step, they were multiplied by 10. In this way, data were scaled in the range (0,1). Next, the logit function was applied (Equation 2) to transform GPRs into real numbers with no upper or lower bounds.

(2) $$\mathrm{logit}\;\left(x\right)=\;\ln\left(\frac{x}{1-x}\right)$$

After applying the logit function, SVR was run on these real numbers and made predictions based on these numbers. Following this step, the numbers were transformed back to a percentage by using the inverse logit function (Equation 3).

(3) $$\frac{\exp\left(\mathrm{logit}\left(x\right)\right)}{1+\exp\left(\mathrm{logit}\left(x\right)\right)}$$

**FIGURE 3:**
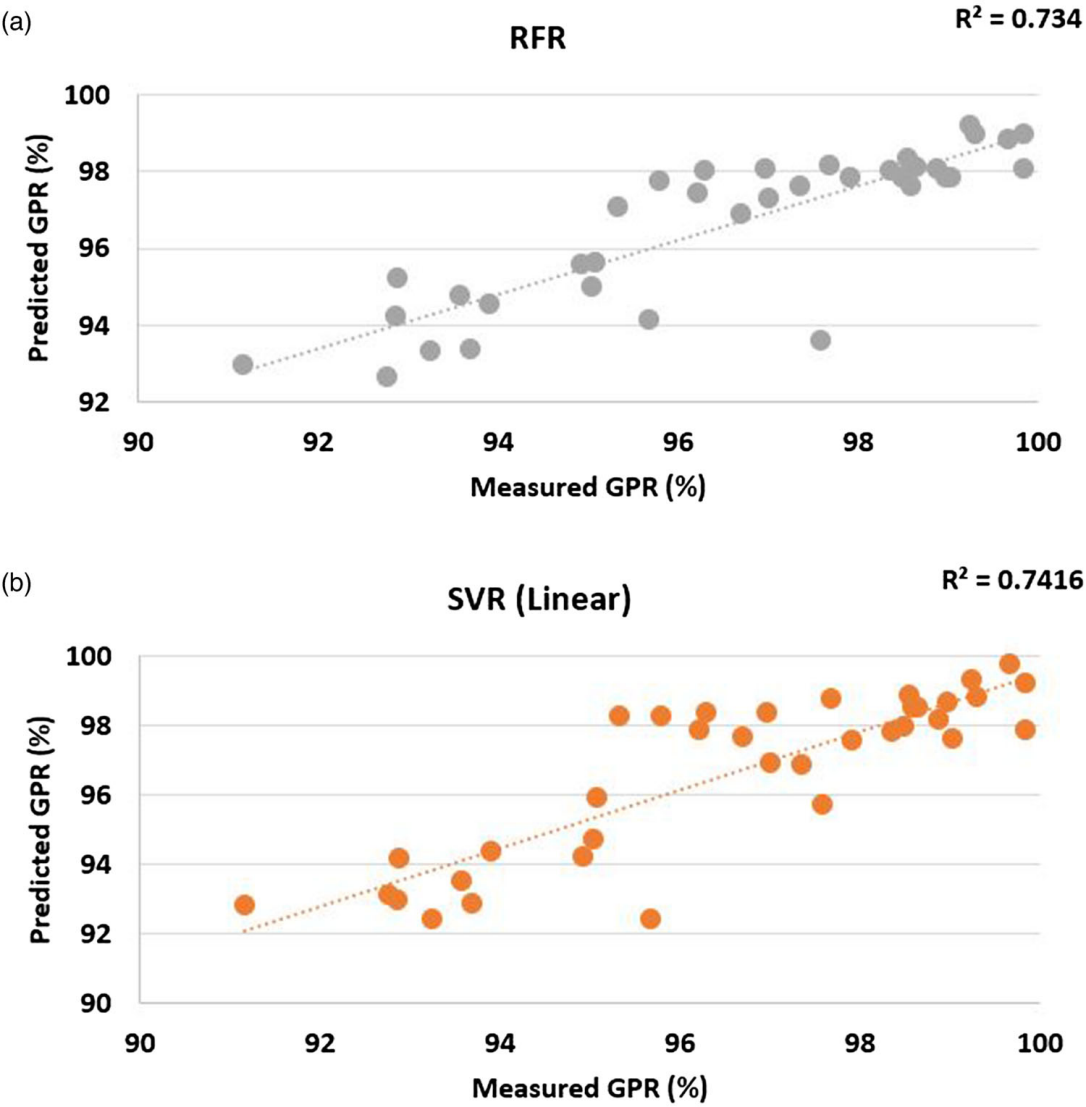
An example of a scatter plot of predicted gamma passing rate (GPR) against measured GPR (3%/2 mm) values for one of the train/test sets, (a) random forest regression, (b) support vector regression (linear)

For evaluation, both models were executed to a test set (36 treatment plans from different sites) that were not used during model development. For RFR, the accuracy was 0.91 with a standard deviation of 0.05, MAE = 1.14 ± 0.084, RMSE equals 1.407 ± 0.103, and the maximum error was 3.494% (Figure 3a). SVR with linear kernel had comparable MAE and RMSE to the RFR model, and MAE was 1.101 ± 0.09 and RMSE equals 1.447 ± 0.121 with a maximum error of 3.424% (Figure 3b). Moreover, *R* ^2^ was equal to 0.703 ± 0.027 and 0.689 ± 0.053 for RFR and SVR, respectively.

For both models, 2 out of 36 treatment plans had a percentage error greater than 3% indicating 94.44% of predictions within 3% of measured 3%/2 mm of GPR.

**FIGURE 4:**
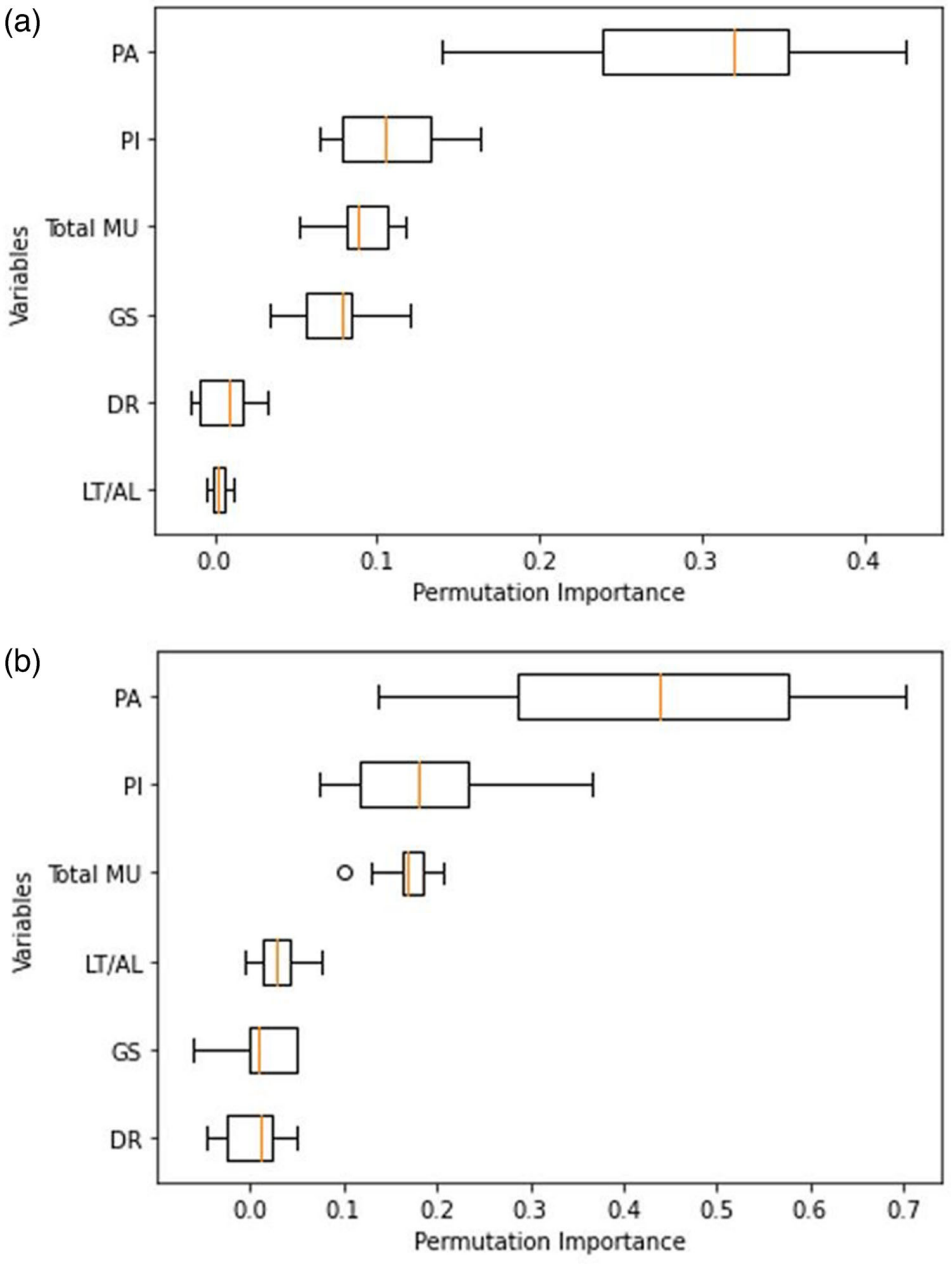
Importance of each variable on gamma passing rate (GPR) prediction accuracy: (a) random forest regression (RFR), (b) support vector regression (SVR) (linear)

The features that were chosen to build the model were ranked with their respective importance for both models (Figure 4). Despite the differences between models, they both determined similar results. Plan‐averaged beam area (PA) has the most fluctuations among all MIs, which is a good indication of different geometry of target across all treatment sites, plus it has a significant impact on the prediction of GPR, whereas DR and LT/AL have the minimal effects on GPR. Overall, PA, PI, and total MU were found to be useful variables to predict the GPRs.

## 4 DISCUSSION

This study has investigated the impact of the combination of plan complexity features on GPR using four treatment sites with variable levels of complexity and on two linacs with different MLC designs. Additionally, we included two VMAT‐specific features GS and DR variations as independent variables to build our model. Generally, MIs are known as important indicators to predict GPR reported by different studies.^7^, ^12^, ^34^, ^35^

Two supervised learning methods were implemented (SVR using linear kernel and RFR) to analyze the data and predict the passing rates. The predicted GPR by the RFR model and SVR model versus measured GPR was plotted (Figure 3). When there is a good agreement between predicted and measured doses, the points form a diagonal line on the graph. However, in practice, the predicted GPR values deviated slightly from the measured values, even though linearity was observed between them. In the present study, 34 out of 36 treatment plans had a percentage error less than 3% in both models, and maximum error was 3.494% and 3.424% for RFR and SVR (linear), respectively. This demonstrates that it is possible to predict passing rates within a 3% error using an SVR with linear kernel and RFR models that combines different complexity metrics. It also indicates that the accuracy of SVR with linear kernel is comparable with RFR model to predict GPR values. This is a valuable improvement over approaches that only analyze the correlation between complexity metrics and QA passing rates individually.

Both RFR and SVR (linear) were in good agreement, and none of their prediction values was greater than 100% (Figure 3). Several studies^11^, ^12^ have shown that RFR has a high level of predictive accuracy, and the complex relationships between the input variables can be modeled. It is considered comparatively robust according to the outliers because the final prediction is the average overall predictions from DTs. Contrary to other ML models, such as artificial neural networks, RF does not overfit data because it randomly samples data from the original dataset and builds multiple DTs, one for each random sample of data.^36^ This way there is a low chance that one DT can see all data.

Our study also demonstrates that the RFR model can predict GPR within a 3% error, which is in good agreement with Bozhikov et al.^11^ and Lam et al.^12^ findings.

To the best of our knowledge, this study is the first to use SVR with linear regression to predict passing rate values; therefore, there is not much information regarding the use of this model. Nevertheless, many studies utilized SVR with the linear kernel for response prediction in another area. In 2016, Goli et al.^31^ employed SVR with different kernels to predict survival time for breast cancer patients. They showed that linear SVR can predict survival time more accurately than nonlinear SVR. Zhang et al.^37^ implemented two SVR (RBF) and SVR (linear) to track tumor motion in the liver. Their results indicated linear SVR predictions were more accurate compared to RBF SVR. Our data also presented the accuracy of SVR (linear) are comparable with RFR and can be useful to predict GPR values.

As was demonstrated in Figures 1 and 2, among all treatment sites, IMRS has the lowest GPR, whereas the prostate has the highest passing rate and lowest fluctuation in MIs. This can be related to the geometrical shape of the prostate target, which is typically spherical without much variation; however, IMRS cases are considered to be more complex as the multitarget shapes are more irregular with numerous OARs surrounding the targets. Figure 4 depicts that PA has the greatest impact on how accurately GPR can be predicted, followed by PI and total MU for both models. The strong correlation between PA and GPR (0.723, *p* < 0.001) demonstrates the effect of field size on GPR. In other words, a large field size can lead to an increased passing rate compared to small field sizes, which is in‐line with prior studies.^10^, ^18^, ^34^ Moreover, it is noted that PI has a negative strong correlation with passing rate (−0.672, *p* < 0.001). As it is shown in Figure 2b, PI values were generally higher in IMRS treatment plans compared to other treatment sites. A main reason is the ability of inverse treatment planning to divide fields in any of the gantry positions to many subfields. This allows the algorithm ability of further optimization as it would have more degrees of freedom to create conformal plan. However, this approach has several drawbacks, such as increased uncertainties in calculations, as well as delivery and dosimetry imperfections. Conversely, increased PI causes lower GPR. Moreover, MU has a strong negative correlation with GPR (−0.66, *p* < 0.001) indicating higher MU results in lower GPR, which agrees with Wu et al.^38^ and Salari et al.^39^ findings.

Additionally, we noted that LT/AL has minimal effect on predicting passing rate which is also shown that it has weaker correlation with GPR. Although strong and moderate correlations were observed between GPR with GS and DR, respectively, these two metrics have low impact on the accuracy of predicting passing rate (Figure 4). PA, PI, and total MU can demonstrate the level of complexity for each treatment site; therefore, the quantification of these parameters may be useful in guiding VMAT plan evaluation and ultimately reducing radiation dose uncertainties.

We acknowledge that there were certain limitations in the current study because the model was created based on our clinical data, a limited number of cases, and using one TPS. There are always some levels of differences between different TPSs and measurement devices, which can affect the output. Therefore, multi‐institutional studies need to be conducted to include all possible differences.

## 5 CONCLUSION

The relationship between GPR (3%/2 mm) and the complexity of VMAT plans was studied by analyzing different MIs. We found that PA, PI, and total MU have the highest level of impact on GPR. However, because MU is already included in PA and PI calculations these metrics may not be fully independent predictors of GPR. According to our result, the ML approach is a useful tool to help medical physicists better evaluate and identify the sources of error in patient‐specific QA measurements.

## AUTHOR CONTRIBUTIONS

Elahheh Salari: conceived and designed the analysis, collected the data, contributed data, performed the analysis, wrote the paper.Kevin Shuai Xu: conceived and designed the analysis, wrote the paper.Nicholas Niven Sperling: conceived and designed the analysis. E. Ishmael Parsai: conceived and designed the analysis, wrote the paper.

## CONFLICT OF INTEREST

The authors declare that there is no conflict of interest that could be perceived as prejudicing the impartiality of the research reported.
